# Dual-Branch Fusion Transformer Networks for Remote Parkinson’s Disease Screening via Hand-Drawn Graphics

**DOI:** 10.3390/bioengineering13101101

**Published:** 2026-09-23

**Authors:** Tzu-Wei Lin, Guan-Jen Li, Wei-Cheng Lien, Yan-Tsung Peng, Yi-Ting Lai, Yu-Hsiu Chen

**Affiliations:** 1 Hyson Technology Inc., New Taipei City 23553, Taiwan; weiweilin886@gmail.com (T.-W.L.); kate920605@gmail.com (G.-J.L.); lien@hysontech.tw (W.-C.L.); 2 Department of Computer Science, National Chengchi University, Taipei 116011, Taiwan; ytpeng@cs.nccu.edu.tw; 3 R&D Center of Biochemical Engineering Technology, Center for Plasma & Thin Film Technologies, Center for Environmental Sustainability and Human Health, Ming Chi University of Technology, New Taipei City 243303, Taiwan; 4 Department of Materials Engineering, Ming Chi University of Technology, New Taipei City 243303, Taiwan; 5 Division of Rheumatology, Immunology and Allergy, Department of Internal Medicine, Tri-Service General Hospital, National Defense Medical University, Taipei 114202, Taiwan; 6 Division of Rheumatology, Immunology and Allergy, Department of Medicine, School of Medicine, College of Medicine, National Defense Medical University, Taipei 114202, Taiwan

**Keywords:** Parkinson’s disease, handwriting recognition, medical information systems, deep learning, image classification

## Abstract

Parkinson’s disease (PD) is one of the most common neurodegenerative disorders in the aging population, which is commonly associated with motor abnormalities that can be reflected in hand-drawn geometric patterns. However, early detection remains challenging due to the lack of specific screening or tests, especially in rural areas with limited medical resources, leading to delays in medical intervention. To address these critical limitations, this paper proposes FusionNet, a dual-branch vision Transformer framework that combines spatial and frequency-domain information for handwriting-based PD screening. The spatial branch learns geometric and stroke-related representations directly from grayscale drawings, whereas the frequency branch processes log-scaled amplitude spectra obtained by two-dimensional fast Fourier transform. Features from the two branches are integrated using bidirectional cross-attention (BCA), allowing spatial and spectral representations to condition one another before classification. A staged training strategy was adopted in which the individual branches were trained separately before optimization of the fusion components. Experimental evaluations on the publicly available NewHandPD dataset were conducted across the three drawing tasks: hand-drawn circle, meander, and spiral images from 66 participants (31 individuals with PD and 35 healthy controls). Performance was evaluated using five-fold Group K-Fold cross-validation, demonstrating that FusionNet achieves the highest accuracy of 91.68% on the meander task and maintains >85% sensitivity with >88% specificity across all tasks. Because the study used a small single dataset, participant-level separation was not guaranteed for all tasks, and no independent external validation was performed, the reported results should be interpreted as preliminary and dataset-specific. Analysis of model behavior further indicates that spatial-domain predictions could be affected by paper texture, whereas the complementary frequency representation reduces this source of bias. Finally, the model was incorporated into a LINE-based prototype to demonstrate the technical feasibility of remote image submission and automated analysis. This implementation remains a proof of concept and requires external, prospective clinical validation before use in clinical decision-making.

## 1. Introduction

The revolution in modern medical technology and improved quality of life have led to low fertility and low mortality, leading to population ageing worldwide [1,2,3], resulting in rising prevalence of neurodegenerative diseases [4]. Parkinson’s disease (PD) is a progressive neurodegenerative disorder affecting roughly 1% of people over 60 [5], characterized by the degeneration of dopaminergic neurons in the substantia nigra, leading to motor symptoms such as resting tremor, bradykinesia, and rigidity [6]. With the global aging population, there is an increasing need for accessible and objective diagnostic tools that can support early detection—particularly since 5–34% of patients experience worsening conditions due to misdiagnosis [7]—and reduce reliance on specialized clinical assessments that are often subjective or difficult to access [8]. The number of people living with PD is increasing worldwide, which has become a global public health issue. Therefore, enhancing accessibility to medical treatment and supportive environments for PD patients is crucial for achieving the aging-related sustainable development goals [9].

Despite the potential benefits of early detection and diagnosis of PD, which facilitate intervention through drug treatments and physical and occupational therapy, approximately 20% of PD patients still face delays or misdiagnosis [7]. These delays result from the lack of a standard conclusive screening or test for the diagnosis of PD. Current clinical diagnosis mainly relies on neurological examination to identify physical signs and symptoms, including bradykinesia, rigidity, and resting tremor [8]. However, such clinical diagnostic criteria often lack specificity, leading to delays or misdiagnosis of PD due to symptom overlap with other disorders, such as multiple system atrophy (MSA) and progressive supranuclear palsy. Besides, delayed diagnosis can increase the risk of developing severe complications, including Alzheimer’s disease (AD), MSA, depression, and even mortality [6]. Blood and other laboratory tests have been reported to detect abnormal α-synuclein and other reliable biomarkers, enabling timely identification of early PD and other synucleinopathies [10]. Neuroimaging techniques, including magnetic resonance imaging (MRI), single-photon emission computed tomography (SPECT), and positron emission tomography (PET), have been reported to increase the accuracy in the diagnosis of PD by evaluating the brain imaging of structural and molecular changes in vivo. However, these laboratory tests and neuroimaging techniques are challenging for routine clinical practice due to their complex features, time-consuming processes, and crowding-out effect on medical resources [11]. Therefore, the development of new technologies for more accurate early-stage assessment of PD is imperative.

Hand-drawn geometric tasks, such as spirals and meanders, have been widely adopted as non-invasive screening methods for PD [12,13]. These tasks require fine motor control and are sensitive to tremor-related abnormalities, making them suitable for capturing subtle motor impairments. Recently, Convolutional Neural Networks (CNNs) have demonstrated strong performance in this domain by effectively extracting spatial features, including line irregularities and stroke-level distortions [14,15], which are indicative of PD-related motor dysfunction [12]. More recently, Ali et al. proposed ChiGa-Net, combining χ^2^ score-based deep feature refinement extracted from handwriting images using genetically optimized neural networks, which addresses concerns regarding overoptimistic performance estimates and cross-dataset generalizability [13]. However, these works are vulnerable to environmental variations and lack model generalizability [15,16], which makes the techniques impractical in real clinical diagnosis. In this work, we introduce FusionNet, a dual-branch architecture designed to harmonize spatial representations with frequency-domain dynamics. The proposed framework utilizes a SpatialNet branch to characterize high-level geometric features and a FreqNet branch to analyze amplitude spectra derived via 2D Fast Fourier Transform (FFT). It enables assessment of hand-drawn spirals in both spatial and frequency characteristics as a diagnostic tool for PD. A Bidirectional Cross-Attention (BCA) mechanism is implemented to facilitate the exchange of information between these domains, enabling the model to capture the synergistic dependencies between structural geometry and spectral power distributions. FusionNet’s contribution lies in integrating spatial and frequency-domain handwriting features through bidirectional cross-attention. Unlike conventional concatenation or late fusion, this enables reciprocal feature conditioning between branches. Experiments on NewHandPD demonstrate strong PD classification performance. FusionNet was also deployed through a cloud-based LINE application for automated screening from uploaded drawings, demonstrating a technical proof-of-concept workflow for remote image submission and automated model inference for PD screening.

To sum up, this work aims to investigate whether complementary spatial- and frequency-domain representations of hand-drawn geometric patterns can improve PD classification. It addresses the limited integration of drawing geometry and spectral characteristics in existing approaches. We hypothesize that reciprocal fusion of these representations can improve task-specific PD classification compared with either representation alone. The proposed framework is intended as a preliminary decision-support approach for accessible remote screening. Note that its clinical utility requires prospective external validation.

## 2. Materials and Methods

### 2.1. Dataset and Synchronous Data Augmentation

This study uses the publicly available NewHandPD dataset [17], which contains handwriting samples from 66 participants, including 31 individuals diagnosed with PD and 35 neurologically healthy controls. All participants completed three standardized drawing tasks: four spiral traces, four meander traces, and one circle trace as shown in Figure 1.

Each task requires the participant to trace a predefined geometric pattern. In the circle task, participants trace a continuous circular template. The spiral task consists of following a curve whose radius progressively increases from the center toward the outer region. The meander task requires the participant to trace a repetitive waveform between a series of parallel vertical guides. Because these patterns impose different requirements on fine motor coordination and trajectory control, they provide complementary representations of deviations in drawing geometry that may be associated with motor impairment.

To address the limited number of available samples and improve model robustness, data augmentation was applied to increase the diversity of image orientations encountered during model training and reduce sensitivity to image acquisition conditions. A Double-Domain Synchronous Augmentation strategy was applied [18]. Importantly, geometric augmentation was performed on the spatial-domain images before frequency-domain transformation. Each image was rotated at 30° intervals, producing 12 orientations ranging from 0° to 330°. A horizontal reflection was generated at each orientation. Rotation augmentation was introduced to account for variations in paper placement and camera or scanner orientation, while horizontal reflection provided geometric regularization to reduce dependence on a particular left-right orientation. However, since reflection may alter drawing chirality, its label-preserving validity was not evaluated. Importantly, these transformations do not create new biological subjects; the dataset contained only 66 independent participants, and augmentation was therefore used solely for geometric regularization rather than as evidence of increased cohort diversity.

### 2.2. Dataset Preprocessing and Frequency Domain Transformation

All hand-drawn images were processed using the same preprocessing procedure. For the spatial-domain branch, images were resized to 256×256 pixels and converted to grayscale, yielding a single-channel representation of the drawing. This representation was used directly as the input to SpatialNet. For the frequency-domain branch, a Two-Dimensional Fast Fourier Transform (2D-FFT) was applied offline to each preprocessed image [19]. For held-out evaluation images, no geometric augmentation was performed, and the 2D-FFT was calculated directly from the resized grayscale image. Accordingly, the actual preprocessing sequence for training samples was resize and grayscale conversion, geometric augmentation, 2D-FFT calculation, log-magnitude transformation, and normalization. The FFT was therefore recalculated after augmentation rather than applying geometric transformations to a precomputed FFT magnitude image. The 2D-FFT converts the image from its spatial representation, I(x, y), into the frequency domain, F(u, v), according to:

(1) $$F\left(u,v\right)=\sum_{x=0}^{M-1}\sum_{y=0}^{N-1}I\left(x,y\right)e^{-j2\pi \left(\frac{ux}{M}+\frac{vy}{N}\right)},$$

where M and N denote the image dimensions (256×256 in this study), j is the imaginary unit, and I(x, y) is the pixel intensity at the spatial coordinate (x, y). The magnitude spectrum was then computed and compressed via logarithmic scaling:

(2) $$S\left(u,v\right)=20\cdot \left(\left|F\left(u,v\right)\right|\right).$$

This operation reduces the dynamic range of the spectral representation, enhancing the discriminability of micro-tremor signatures for the subsequent feature extraction layers.

### 2.3. Normalization

To ensure numerical stability and mitigate the risk of gradient explosion within the dual-branch architecture, Z-score normalization was applied to all inputs:

(3) $$z=\frac{x-\mu }{\sigma }.$$

Normalization parameters were computed empirically from each cross-validation fold’s own training data, separately for the spatial-domain and frequency-domain (FFT-transformed) images.

### 2.4. Development of Integrative AI Model

#### 2.4.1. Baseline: Support Vector Machine (SVM)

To benchmark classification performance, an SVM with a Radial Basis Function (RBF) kernel [20] was implemented. Images were resized to 64 × 64 pixels and flattened into 4096-dimensional raw pixel-intensity vectors, rather than using handcrafted or learned features. Within each cross-validation fold, a StandardScaler was fitted exclusively to the training data and then applied unchanged to the corresponding held-out data, thereby preventing preprocessing leakage. The SVM used a penalty parameter of C=1.0, class weights of $W_{HC}=1.5$, $W_{PD}=1.0$ and scikit-learn’s default γ=“scale”, defined as $\frac{1}{\left(n_{features\;}\times Var\left(X_{train}\right)\right)}$. Neither C nor γ was optimized through grid search or another systematic tuning procedure. Thus, the SVM served as a fixed conventional baseline for evaluating the discriminative potential of standardized raw pixel intensities without spatial–frequency fusion, rather than as a matched-complexity comparison with the transformer-based models, which used 256 × 256 inputs.

#### 2.4.2. Proposed Method: Bidirectional Cross-Attention Transformer

Figure 2 shows the proposed dual-branch (FusionNet). The dual-branch network adopts Bidirectional Cross-Attention Transformer to facilitate feature fusion between spatial and frequency domain representations. The model consists of two symmetric transformer-based single-branch branches, denoted as SpatialNet and FreqNet.

#### 2.4.3. Dual-Branch Feature Extraction

Each branch begins with a patch embedding layer employing a 3×3 convolution with a stride of 1 to project the input into a high-dimensional feature space while preserving local structural continuity. The resulting feature maps are subsequently refined through a stack of Transformer blocks, each consisting of a Multi-DConv Head Transposed Self-Attention (MDTA) module followed by a Gated Depth-wise Convolutional Feed-Forward Network (GDFN), with layer normalization and residual connections applied at each stage [21]:

(4) $$x=x+MDTA\left(LN\left(x\right)\right),\;x=x+GDFN\left(LN\left(x\right)\right).$$

#### 2.4.4. Multi-DConv Head Transposed Self-Attention (MDTA)

The MDTA module facilitates global interaction by computing the attention map across the channel dimension. The Query (Q), Key (K), and Value (V) are generated by applying a 1 × 1 pointwise convolution followed by a 3 × 3 depth-wise convolution to encode local context. The attention is computed as:

(5) $$Attention\left(Q,K,V\right)=Softmax\left(QK^{T}\cdot \tau \right)V,$$

where τ is a learnable temperature parameter used to scale the dot product.

#### 2.4.5. Gated Depth-Wise Convolutional Feed-Forward Network (GDFN)

The GDFN refines features through a gating mechanism to suppress noise and emphasize tremor patterns. The input is projected to twice the hidden dimension via a 1 × 1 convolution, processed by a 3 × 3 depth-wise convolution, and split into two parallel paths, one of which passes through a GELU activation function (Φ) [22]:

(6) $$GDFN\left(Z\right)=W_{out}\left(\Phi \left(DWConv{\left(W_{in}Z\right)}_{1}\right)⊙DWConv{\left(W_{in}Z\right)}_{2}\right),$$

where $W_{in}$ and $W_{out}$ denote the input and output pointwise projections; ⊙ denotes the Hadamard product and DWConv represents depth-wise convolution.

#### 2.4.6. Bidirectional Cross-Attention Fusion

To facilitate deep interaction between the spatial and frequency domains, a BCA mechanism is employed, enabling mutual information exchange between SpatialNet and FreqNet. Two independent cross-attention modules are applied in parallel. In the first, spatial features serve as Q while frequency features provide K and V, capturing the alignment of stroke geometry with spectral pattern representation ${out}_{1}$. In the second, the roles are reversed, allowing frequency features to attend to spatial context, producing ${out}_{2}$. The fused representation $Z_{fused}\;$ is obtained via element-wise summation of the two outputs:

(7) $$Z_{fused}={out}_{1}+{out}_{2}.$$

#### 2.4.7. Feature Refinement and Gating

Following the BCA fusion process, the fused representation $Z_{fused}$ is subsequently passed through an additional Transformer block. This refinement step enables the network to reorganize and consolidate the complementary spatial- and frequency-domain information obtained from the preceding cross-attention operation. The resulting feature map therefore incorporates information exchanged between the two branches rather than relying on either representation independently. Global Average Pooling (GAP) is then applied to aggregate spatial information into a compact feature vector, followed by a linear classifier with the Softmax activation:

(8) $$Y=Softmax\left(Linear\left(GAP\left(Z_{final}\right)\right)\right).$$

This refinement-and-classification stage transforms the cross-domain feature representation into the final prediction while retaining information jointly learned from the spatial and frequency branches.

#### 2.4.8. Three-Stage Training Strategy

A progressive three-stage training procedure was adopted to promote stable learning of the spatial- and frequency-domain representations before their integration in FusionNet. As shown in Figure 2, the two branches were first optimized independently and were subsequently incorporated into the dual-branch architecture, thereby stabilizing training and preserving domain-specific representations before cross-domain fusion.

Stage 1: The SpatialNet branch is trained independently on raw spatial-domain images. This decoupled phase allows the network to focus exclusively on extracting complex geometric patterns and structural distortions without cross-domain gradient interference.

Stage 2: The FreqNet branch is trained independently on log-scaled 2D-FFT amplitude spectra.

Stage 3: The weights obtained after Stage 1 and Stage 2 training are loaded into their respective branches and fully frozen to preserve domain-specific knowledge. Training is then restricted entirely to the BCA modules, the feature refinement Transformer block, and the final classification head.

Throughout Stages 1 and 2, both networks are optimized using AdamW [23] with a weight decay of $10^{-4}$ and a cosine annealing learning rate schedule [24] to achieve stable convergence. This freeze-and-fuse scheme preserves the learned representations while enabling effective cross-domain interaction modeling. The BCA modules adaptively regulate the contribution of spatial and spectral features, facilitating their integration and improving overall diagnostic performance.

### 2.5. Training and Evaluation Protocol

#### 2.5.1. Experimental Settings

All experiments were implemented in PyTorch 2.10.0+cu128 with CUDA 12.8 and conducted on a workstation equipped with an NVIDIA GeForce RTX 4090 GPU (NVIDIA Corporation, Santa Clara, CA, USA). Within each cross-validation fold, each training stage was run for 20 epochs with a batch size of 8. AdamW was used with an initial learning rate of $1\times 10^{-4},$ weight decay of $1\times 10^{-2}$, cosine annealing to $\eta_{min\;}=1\times 10^{-5}$. Gradient-norm clipping was applied with a maximum norm of 1.0. No fixed random seed was specified using torch.manual_seed() or np.random.seed(); therefore, exact run-to-run reproducibility is not guaranteed. The learning rate and batch size were selected empirically, while the embedding dimension, Transformer blocks, and attention heads followed the Restormer-style backbone design. No systematic hyperparameter optimization was performed; therefore, this configuration is not claimed to be optimal.

#### 2.5.2. Computational Requirements

For 256×256 inputs, a single branch required 14.15 GMACs (~28.3 GFLOPs), whereas FusionNet required 33.62 GMACs (~67.2 GFLOPs). On an NVIDIA GeForce RTX 4090, batch-1 inference latency was 6.0 ms/image for a single branch and 14.5 ms/image for FusionNet. At batch size 8, peak inference memory was 2.56 and 2.70 GB, respectively. Peak training memory was 18.22 GB for a fully trainable single branch and 7.32 GB for FusionNet with both branches frozen. Based on measured batch times, estimated per-epoch training times were 36–142 s for a single branch and 32–129 s for FusionNet, depending on the task. These hardware-specific measurements exclude preprocessing, data transfer, and network latency.

#### 2.5.3. Model Complexity

SpatialNet and FreqNet each contained 217,010 parameters, while the complete FusionNet contained 515,646 parameters. During Stage 3, both branches were frozen; thus, only 81,626 parameters in the BCA, refinement, and classification components remained trainable.

#### 2.5.4. Performance Metrics

Five-fold Group K-Fold cross-validation was used for model evaluation. For the circle task, participant identity was used as the grouping variable. For the meander and spiral tasks, the original drawing instance was used as the grouping variable, such that all augmented variants derived from the same source drawing remained within the same fold. Therefore, participant-level separation was ensured for the circle task but not for the meander and spiral tasks. The latter results should consequently be interpreted as drawing-level, dataset-specific performance estimates. Samples were partitioned using the Group K-Fold with subject ID, ensuring that all original and augmented images from each participant were assigned to the same fold. In each iteration, four folds were used for training, and the remaining fold was reserved for testing. Each original training image was augmented into 24 variants, corresponding to 12 rotation angles, with and without horizontal reflection, whereas only the original, non-augmented images were used for evaluation in the held-out fold. For the circle task, training-set sizes across folds 1–5 were 1248, 1272, 1272, 1272, and 1272 images, respectively, while the corresponding test sets contained 14, 13, 13, 13, and 13 held-out images, respectively. For the meander task, the corresponding training-set sizes were 5064, 5064, 5064, 5064, and 5088 images, with 53, 53, 53, 53, and 52 held-out images. This subject-level partitioning allows augmented variants of the same drawing to be kept within the same fold. Model performance was evaluated using accuracy, F1-score, precision, recall (sensitivity), and specificity, as defined in Equations (9)–(13):

(9) $$Accuracy=\frac{TP+TN}{TP+TN+FP+FN}\;\times 100\%,$$

(10) $$F1-Score=\frac{2}{{Recall}^{-1}+{Precision}^{-1}}\times 100\%,$$

(11) $$Precision=\frac{TP}{TP+FP}\times 100\%,$$

(12) $$Recall=Sensitivity=\frac{TP}{TP+FN}\times 100\%,$$

(13) $$Specificity=\frac{TN}{TN+FP}\times 100\%,$$

where *TP* represents true positives, *FP* represents false positives, *TN* represents true negatives, and *FN* represents false negatives. Accuracy reflects the overall proportion of correctly classified samples, whereas precision evaluates the reliability of positive predictions. Sensitivity measures the ability to correctly identify PD cases and is particularly important for screening applications, where minimizing false negatives is essential. Specificity quantifies the capability to correctly identify healthy individuals and avoid unnecessary medical referrals.

Performance metrics were calculated separately for each held-out fold and reported, for each task and model, as mean ± standard deviation across the five folds. Results were not pooled across the circle, meander, and spiral tasks. Standard deviation represents fold-to-fold variability rather than a confidence interval. Because only five folds were available and their training sets overlapped, 95% confidence intervals and formal pairwise statistical tests were not performed; therefore, model comparisons are descriptive. Note that, for training, no separate validation set or early stopping was used. Within each fold, training continued for 20 epochs, and the checkpoint yielding the highest accuracy on the evaluation fold was retained. Precision, recall, F1-score, and specificity from the same epoch were used as that fold’s final metrics and summarized across the five folds. Because the held-out fold was used for both checkpoint selection and performance estimation, the reported results may be optimistically biased.

## 3. Results

### 3.1. Comparison Between Single-Branch and Dual-Branch Networks

Table 1 shows the classification performance of SpatialNet, FreqNet, and the proposed FusionNet across the circle, meander, and spiral tasks. Overall, FusionNet outperforms both single-branch models across all tasks and metrics, demonstrating the complementary nature of spatial and frequency-domain representations. It is noted that the meander task achieves the highest performance, for which FusionNet achieved an accuracy of 91.68%, an F1-score of 91.66%, a precision of 91.68%, a sensitivity of 91.85%, and a specificity of 90.02%. Compared with FreqNet and SpatialNet, the specificity improved by 3.63% and 30.16%, respectively. For the circle and spiral tasks, FusionNet also maintained sensitivity above 85% and specificity above 88%, indicating that the combined representation provided comparatively consistent discrimination between PD and healthy-control samples across different drawing patterns.

Interestingly, the single-branch FreqNet consistently exhibits considerably higher specificity than SpatialNet, particularly for the meander and spiral tasks. For example, in the spiral task, FreqNet achieved a specificity of 85.03%, compared with 59.86% for SpatialNet. This difference suggests that frequency-domain representations are inherently less sensitive to local image variations and background artifacts. Unlike spatial features that rely heavily on pixel intensity distributions, spectral representations primarily capture the periodic fluctuations induced by motor instability, thereby improving robustness against environmental disturbances. These findings indicate that spatial and frequency-domain features contain complementary information. While the spatial branch emphasizes geometric irregularities and stroke distortions, the frequency branch captures tremor-related spectral characteristics. The results indicate that integrating spatial- and frequency-domain representations within the proposed FusionNet framework provides a more balanced classification profile than either representation alone. Because alternative fusion strategies were not evaluated, the incremental contribution of BCA itself cannot be determined from the present experiments.

The FreqNet is used to transform images to the frequency domain using an FFT technique. Herein, we converted the signal into sine and cosine components, then removed the imaginary parts of the sine components. The resulting frequency spectrum enables a CNN model to distinguish input spiral images from non-PD individuals and PD patients. For example, the spiral diagrams from PD patients display significant noise and overlapped circular patterns, reflecting irregular frequency characteristics (Figure 3a). These irregularities prompt the AI to concentrate on high-frequency domains. Conversely, spirals drawn by normal individuals typically consist of regular circular patterns, leading the AI to focus on low-frequency domains (Figure 3b). This explains why FreqNet exhibits significantly higher specificity than SpatialNet. The proposed FusionNet effectively utilizes the frequency characteristics of the hand-drawn diagrams to enhance the accuracy of PD diagnosis.

### 3.2. Practical Implications of AI-Assisted Remote PD Diagnosis System

Building upon the proposed AI-assisted handwriting-based PD classification technique, we develop a preliminary remote-screening workflow by integrating the model with the LINE application (LY Corporation, Tokyo, Japan), as illustrated in Figure 4. This implementation was designed to demonstrate the technical feasibility of receiving user-submitted handwriting images through a commonly used communication platform and processing them using the proposed AI framework. A demonstration video (Video S1) presents the recommended procedure for capturing and uploading handwriting images through the LINE-based interface.

During implementation, we observed that image transmission through LINE introduced an additional source of variability. Image compression applied during transmission altered image texture and frequency-domain characteristics, which affected the performance of the FFT-based classifier. The specificity for healthy individuals decreased from 86.40% to 45.53% after LINE transmission (Figure 5). The frequency-domain representation was sensitive to image compression during the transmission process. These experimental results indicated a technical limitation of the LINE-based workflow. A previous study from our group independently evaluated a spatial-feature pattern check for LINE-compressed spiral images [25]. That study used a separate small cohort consisting of 12 PD and 11 healthy-control spiral drawings. Low-level spatial features were extracted from ImageNet-pretrained VGG19 and ResNet152 models and subsequently classified using a K-nearest neighbors (KNN) algorithm with K-fold cross-validation. Random image scaling was applied before testing the spatial-feature model using LINE-compressed images. The previous study reported a sensitivity of 78.84% and a specificity above 93% for the spatial-feature module, suggesting a potential route to address the practical limitation of the LINE-based PD screening service.

The prototype combines multiple machine-learning and deep-learning components, image upload through LINE, and an SQL interface to the hospital registration system (Video S2 in Supplementary Material). This integration demonstrates workflow feasibility only. At the current stage, this LINE-based system should be regarded as a proof-of-concept rather than a clinically validated telemedicine platform. Accordingly, its accessibility, affordability, clinical effectiveness, and potential impact on healthcare equity have not yet been established. Further prospective technical testing, usability assessment, clinical validation, and economic evaluation are required before conclusions can be drawn regarding its practical healthcare benefits. The primary functionalities of the AI-based healthcare service are listed as follows:

(1) Remote PD assessment: The service provides a potential tool for fast PD evaluation and report for users, with >85% sensitivity and >88% specificity. Note that these results were obtained under the experimental protocol of this study and should not be interpreted as evidence of clinical effectiveness in real-world settings.
(2) Healthcare education of PD: This system provides information about PD, including its definition, etiology, clinical symptoms, and treatment suggestions. This information is intended for educational purposes and does not replace professional medical consultation or clinical diagnosis.
(3) Integration of hospital registration: The prototype demonstrates integration with a hospital registration system, allowing users to make an appointment with doctors remotely at any time through the LINE-based interface. This implementation demonstrates the technical feasibility of connecting the proposed AI-assisted assessment framework with existing healthcare information systems; however, its usability and effectiveness in routine clinical practice require further evaluation.

## 4. Discussion

This section discusses evaluations for the proposed FusionNet from both performance and interpretability perspectives. First, FusionNet is compared with conventional SVM classifiers using spatial- and frequency-domain features to determine whether hierarchical dual-domain feature learning provides measurable advantages across different drawing tasks. In particular, we focus on the balance between sensitivity and specificity, which is critical for reliable clinical screening. Subsequently, Gradient-weighted Class Activation Mapping (Grad-CAM) analysis is employed to investigate the decision-making behavior of the spatial-domain model and to identify potential texture-induced domain bias caused by variations in paper background. These analyses reveal the vulnerability of single-domain models to shortcut learning and demonstrate how the integration of complementary spatial and spectral representations, together with the BCA mechanism, allows FusionNet to suppress environmentally induced artifacts, preserve disease-relevant motor characteristics, and achieve more balanced, generalizable, and clinically meaningful PD classification.

### 4.1. Comparison with Conventional Machine Learning Models

To further assess the effectiveness of proposed hierarchical feature extraction over conventional machine-learning approaches, FusionNet was compared with Support Vector Machine (SVM) classifiers constructed using spatial-domain and frequency-domain inputs. As shown in Table 2, FusionNet generally achieves superior performance in accuracy, precision, F1-score, and specificity across all three drawing tasks. Although several SVM models exhibit slightly higher sensitivity, their improvements are accompanied by a noticeable reduction in specificity, indicating a tendency toward overdiagnosis. For example, in the circle task, both FreqNet-SVM and SpatialNet-SVM achieve a sensitivity of 93.33%, which exceeds the 85.95% obtained by FusionNet. However, their corresponding specificities are 60.00% and 80.00% for FreqNet-SVM and SpatialNet-SVM, respectively, whereas FusionNet achieves a specificity of 88.57%. Such observations suggest that conventional SVM models tend to classify ambiguous samples as PD-positive. This pattern indicates that assessment based on sensitivity alone may provide an incomplete representation of model performance, particularly for a screening application in which both false-negative and false-positive predictions are relevant.

For the meander task, FusionNet achieves an accuracy of 91.68%, an F1-score of 91.66%, and a specificity of 90.02%, exceeding the corresponding values obtained using either SVM input representation. For the spiral task, though the spatial-domain SVM achieves a slightly higher sensitivity than FusionNet (95.09% versus 91.07%), FusionNet produces a comparable overall classification performance. These results suggest that the dual-branch model does not obtain its performance advantage by maximizing a single evaluation metric, but rather by maintaining a more balanced relationship among sensitivity, precision, and specificity. The superior F1-scores of FusionNet across the meander and spiral tasks also support the better harmonic means of precision and sensitivity.

To minimize leakage of augmented observations across training and test subsets, Group K-Fold cross-validation was applied. Circle samples were grouped according to participant identity, whereas meander and spiral samples were grouped at the level of the original drawing instance, such that all augmented versions derived from the same source drawing were assigned to the same fold. This procedure prevents augmented copies of an individual drawing from appearing simultaneously in the training and test sets. However, because participant-level grouping was not applied to the meander and spiral tasks, drawings obtained from the same participant could potentially occur in different folds. The corresponding results should therefore be interpreted as dataset-specific estimates rather than evidence of participant-independent clinical generalizability. Future studies should evaluate the proposed framework using strictly participant-grouped partitions, larger and demographically diverse cohorts, and independent external datasets.

The relatively consistent performance across multiple metrics suggests that jointly learned spatial and frequency-domain representations may provide more discriminative information than classification based on either input representation alone. Such balanced behavior is particularly desirable for real-world telemedicine systems, where both missed diagnoses and excessive false alarms should be minimized. The results demonstrate that FusionNet can achieve a more balanced and clinically meaningful trade-off between sensitivity and specificity under the experimental conditions used in this study.

### 4.2. Comparison with Previously Published Handwriting-Based PD Detection Approaches

To contextualize the present findings, Table 3 compares FusionNet with representative handwriting-based PD detection approaches. Current work in this area primarily focused on spatial characteristics of drawing trajectories, whereas the present study investigates whether spatial information can be complemented by frequency-domain characteristics within a dual-branch Transformer framework. Alissa et al. [16] developed a relatively compact CNN to classify figure-copying tasks obtained from 87 participants, including 58 patients with PD and 29 healthy controls. Their study analyzed wire-cube and spiral-pentagon drawing tasks by using a digitizing tablet, which showed a maximum classification accuracy of 93.5% for the pentagon drawing task. In comparison, the present FusionNet framework uses static hand-drawn circle, meander, and spiral images from the NewHandPD dataset, which does not rely on graphics tablet-derived information such as pressure or in-air trajectories. The highest accuracy in the present study was 91.68% for the meander task. Although this value is comparable to that reported by Alissa et al., various factors such as drawing tasks, acquisition systems, and cross-validation procedures between the studies should be further assessed.

Li et al. [12] proposed a Continuous Convolution Network (CC-Net) for classification of template-free Archimedean spiral drawings. Their dataset comprised 86 participants, with 43 PD patients and 43 healthy controls, achieving 89.3% accuracy, with an average AUC of 0.934. A key design feature of CC-Net is the reduction of pooling operations to retain fine visual information from hand-drawn trajectories. This objective is conceptually related to the spatial branch of FusionNet, which also aims to preserve local geometric and stroke-related abnormalities. However, FusionNet differs in that it explicitly introduces an additional frequency-domain representation. This allows the model to characterize not only local geometric distortions but also the distribution of image information across spatial frequencies.

More recently, Ali et al. [13] introduced ChiGa-Net that uses $\chi^{2}$-based refinement of deeply extracted features followed by a genetically optimized neural network. ChiGa-Net was trained and validated using NewHandPD and subsequently evaluated using the independent HandPD dataset. During external testing of spiral images, the authors reported a balanced accuracy of 92.56%, sensitivity of 85.13%, and specificity of 100%. Although FusionNet achieved sensitivity above 85% and specificity above 88% within the evaluation protocol used here, present work lies primarily in investigating the feasibility of integrating spatial and domain representations through a dual-branch Transformer architecture.

Overall, the existing literature and the present results suggest that no single representation or architecture has yet established universally superior performance for handwriting-based PD screening. The comparatively strong performance reported across these studies is encouraging, but differences in validation design substantially affect the interpretation of numerical metrics. The proposed FusionNet framework supports further investigation of spatial-frequency fusion for handwriting-based PD classification.

### 4.3. Interpretability Analysis and Texture-Induced Domain Bias

To better understand the origin of misclassification, Grad-CAM visualizations were employed to investigate the attention patterns of the single-branch SpatialNet. A pronounced domain bias was identified. Specifically, HC samples were collected using two different paper types: one subset employed smooth, homogeneous paper, whereas the other contained visible fibrous textures and uneven background illumination. Interestingly, SpatialNet exhibits 100% false positive rate for HC samples using the latter paper type.

In Figure 6a, HC samples recorded on smooth paper, Grad-CAM activation maps exhibit warm orange regions distributed predominantly over the blank background areas rather than the handwriting itself. This observation indicates that SpatialNet inadvertently learns to associate background smoothness or “background purity” with the healthy class. Consequently, environmental characteristics became highly predictive features during training. However, when healthy samples were recorded on rough paper, the activation patterns changed dramatically. As shown in Figure 6b, the warm orange activations disappear and are replaced by cool blue regions. The fibrous paper texture significantly obscures the learned background signature, causing SpatialNet to misclassify these healthy subjects as PD cases. Remarkably, all rough-paper healthy samples were incorrectly predicted, corresponding to a 100% false-positive rate within this subgroup.

The above behavior represents a typical example of shortcut learning [26], where deep neural networks exploit spurious correlations that are statistically associated with class labels but are unrelated to the underlying pathology. Instead of focusing on disease-relevant motor abnormalities, the model relied on superficial environmental cues introduced during data acquisition. Similar phenomena have been widely reported in medical imaging studies [27], where models inadvertently learn hidden data acquisition biases, such as scanner-specific artifacts, image contrast variations, or acquisition settings, rather than genuine biological characteristics [28]. These findings highlight an important challenge in medical AI systems: high classification accuracy alone does not necessarily imply that a model has learned clinically meaningful representations.

In contrast, the proposed FusionNet model adopts both frequency-domain and spatial-domain branches for PD diagnosis. From a signal-processing perspective, paper texture mainly contributes to uniformly distributed high-frequency noise and does not exhibit the structured harmonic patterns generated by handwriting trajectories. The frequency-domain branch can focus on intrinsic motor characteristics rather than localized background variations. Therefore, FreqNet compensates for the texture-induced artifacts learned from SpatialNet, leading to higher specificity across all drawing tasks. Besides, the proposed FusionNet further alleviates shortcut learning through the BCA mechanism. Unlike conventional feature concatenation, BCA enables reciprocal interaction between spatial and spectral representations, allowing each branch to validate information extracted by the other. This mutual verification can suppress spurious correlations and encourages the model to base its predictions on features that are consistently present across both domains. Therefore, the improved specificity observed in FusionNet can be attributed not only to the complementary nature of spatial and spectral representations but also to BCA’s ability to mitigate environmentally induced shortcuts and reinforce genuine motor-related characteristics.

Interestingly, the discovery of texture-induced bias is not regarded as a failure of the proposed framework but rather as evidence of the necessity of multimodal learning. The observed shortcut-learning phenomenon demonstrates that single-domain representations may be vulnerable to environmental confounders, whereas the incorporation of complementary spectral information enables the network to anchor its predictions to intrinsic motor dynamics. Consequently, FusionNet serves not only as a performance-enhancing architecture but also provides a potential avenue of AI-assisted PD screening.

### 4.4. Study Limitations

Several limitations should be considered when interpreting the present findings. (1) The NewHandPD dataset contains only 66 independent participants and represents a single publicly available cohort; therefore, the present study does not establish external generalizability. (2) Augmented variants derived from the same source drawing were retained within the same fold; however, the meander and spiral experiments were grouped according to drawing instance rather than participant identity. Different drawings obtained from the same participant could therefore occur in different folds, potentially resulting in optimistic performance estimates. (3) Geometric augmentation increased image variation but did not increase biological or demographic diversity. (4) The observed influence of paper texture and LINE-mediated image compression demonstrates that image acquisition and transmission conditions can affect model behavior. (5) The present comparisons do not isolate the incremental contribution of BCA relative to simpler fusion strategies. Accordingly, the current findings should be regarded as exploratory and dataset-specific. Future studies should employ strictly participant-grouped evaluation, independent model-selection procedures, larger external cohorts, and prospective testing under standardized acquisition conditions.

## 5. Conclusions

This study proposes FusionNet, a dual-branch transformer-based architecture designed to integrate spatial and frequency-domain features for handwriting-based PD screening. Enabled by BCA, the dual-modal framework achieved sensitivity above 85% and specificity above 88% across the three drawing tasks in the NewHandPD dataset. The results indicate that the integration of spatial and spectral representations can improve the balance between sensitivity and specificity compared with the individual branches. Notably, spatial-domain predictions could be influenced by acquisition-related artifacts, particularly differences in paper texture, which can be mitigated by frequency-domain of FusionNet, highlighting its capability of addressing the shortcut learning and acquisition-related bias for image-based screening models. The proposed framework was additionally incorporated into a LINE-based system to demonstrate the technical feasibility of remote image submission and automated analysis. Although this implementation remains a technical proof of concept and the system has not undergone prospective technical, usability, or clinical validation, these preliminary findings have demonstrated the technical feasibility of remote image submission and automated analysis for PD screening. Future work should prioritize strictly participant-grouped evaluation, larger and more diverse external cohort analysis, and practical testing under realistic remote-screening conditions to establish robustness, generalizability, and clinical utility of this model.

## Figures and Tables

**Figure 1:**
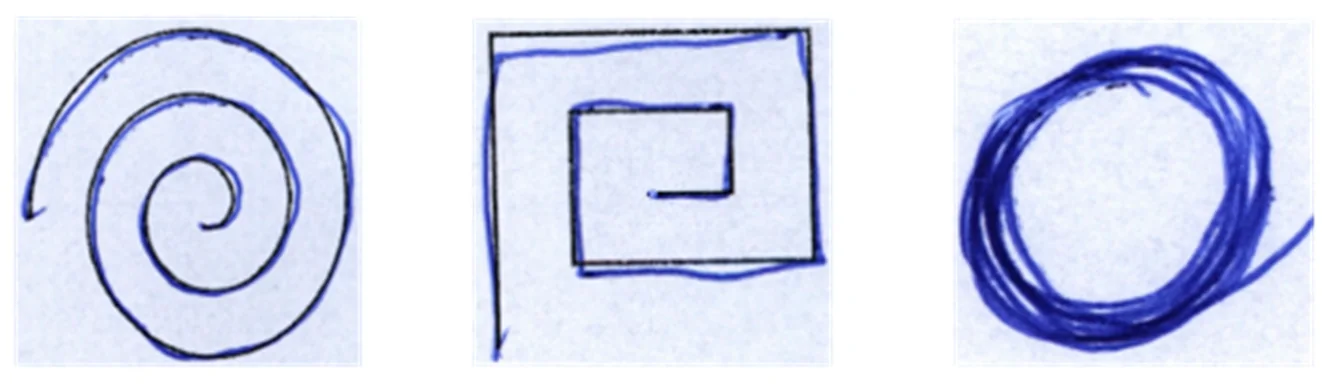
The three hand-drawn geometric templates used in this study, spiral, meander, and circle, are illustrated from left to right, respectively.

**Figure 2:**
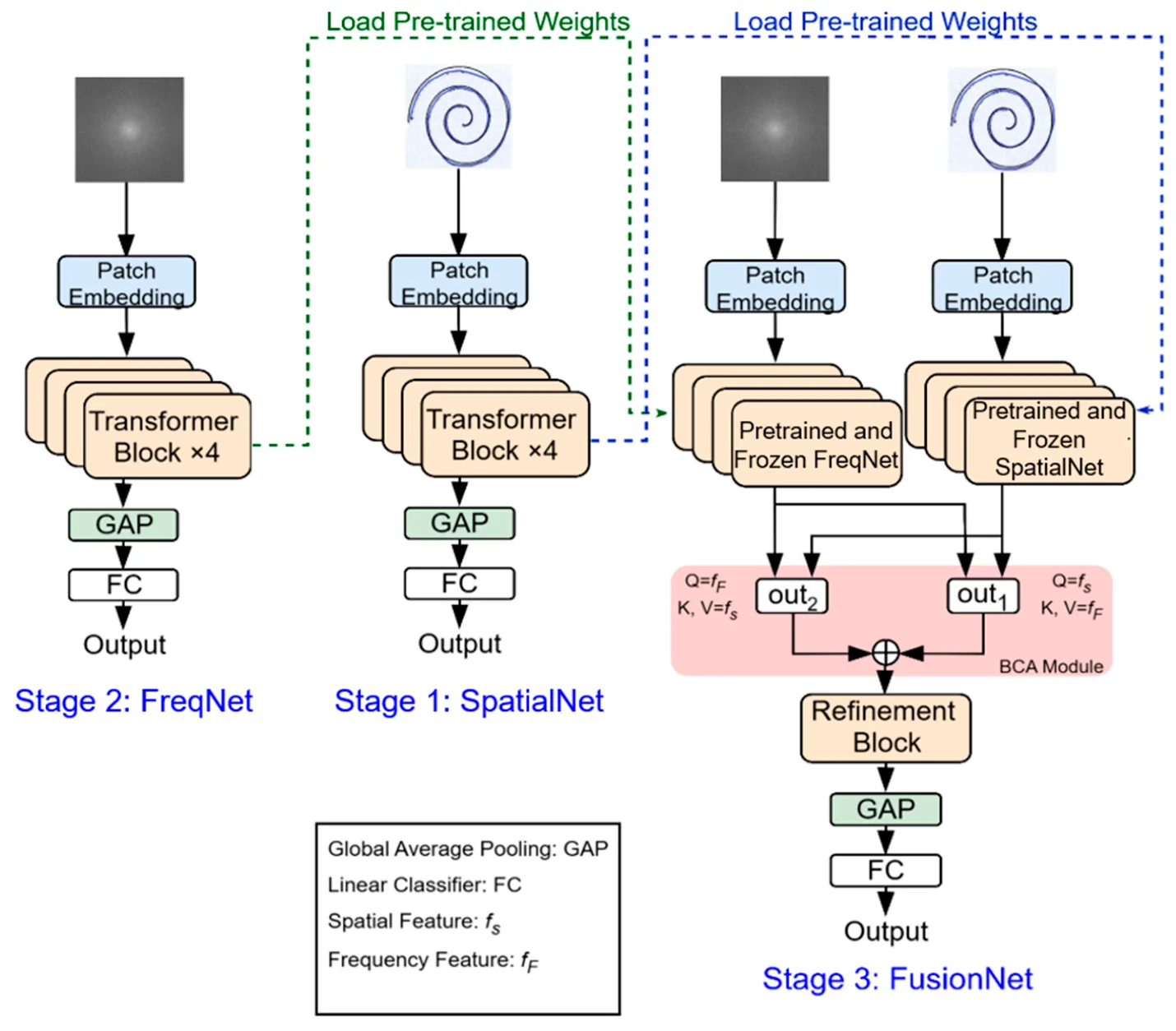
The architecture of the proposed diagnostic framework. The complete FusionNet was integrated with spatial and spectral features through a BCA module.

**Figure 3:**
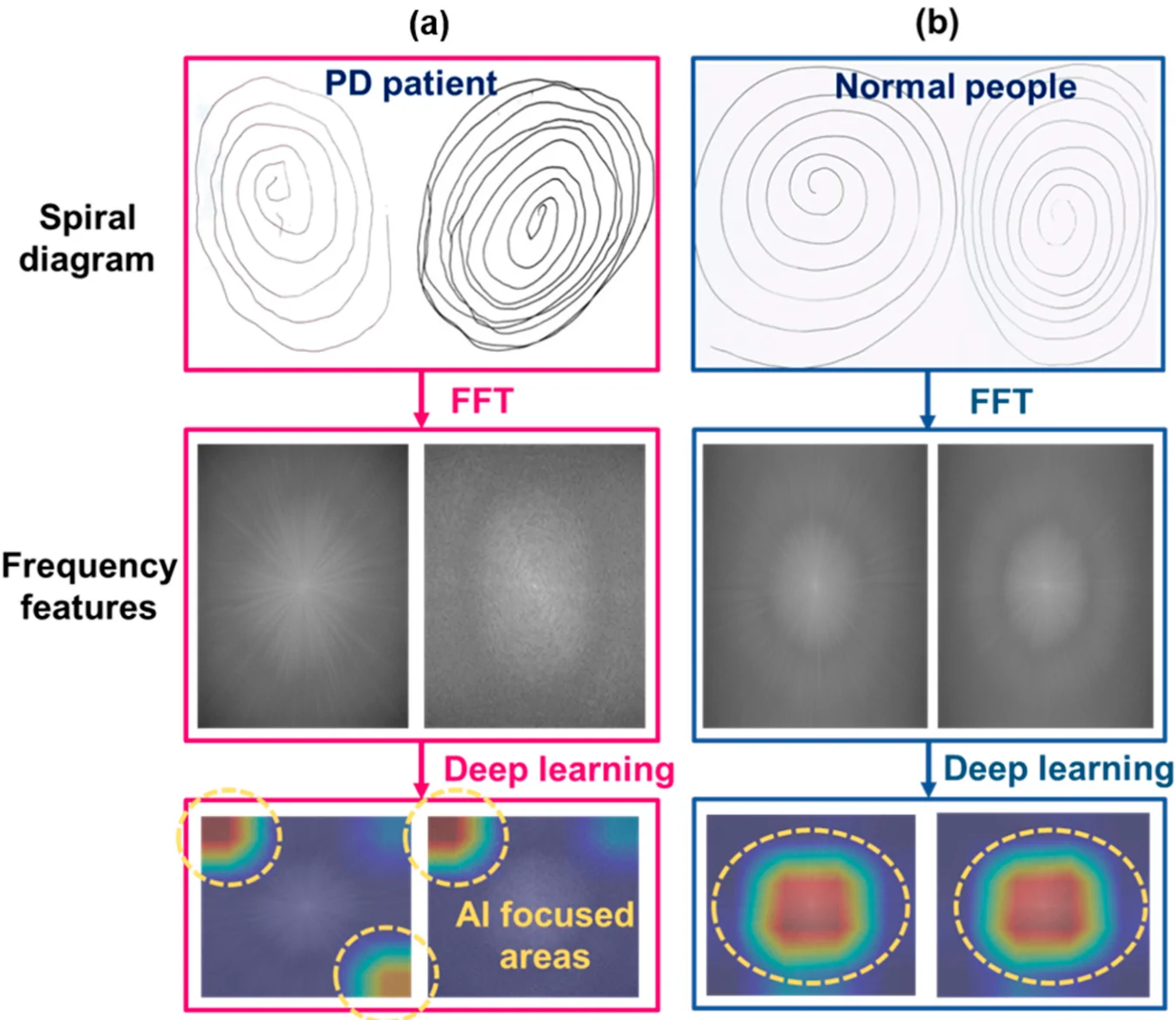
The frequency domain transformation is based on the FFT technique. (**a**) The spiral diagrams from PD patients at different stages show lots of noise, which allows AI to focus on the high-frequency domains, while (**b**) those from normal people contain regular circles, which allows AI to focus on the low-frequency domains.

**Figure 4:**
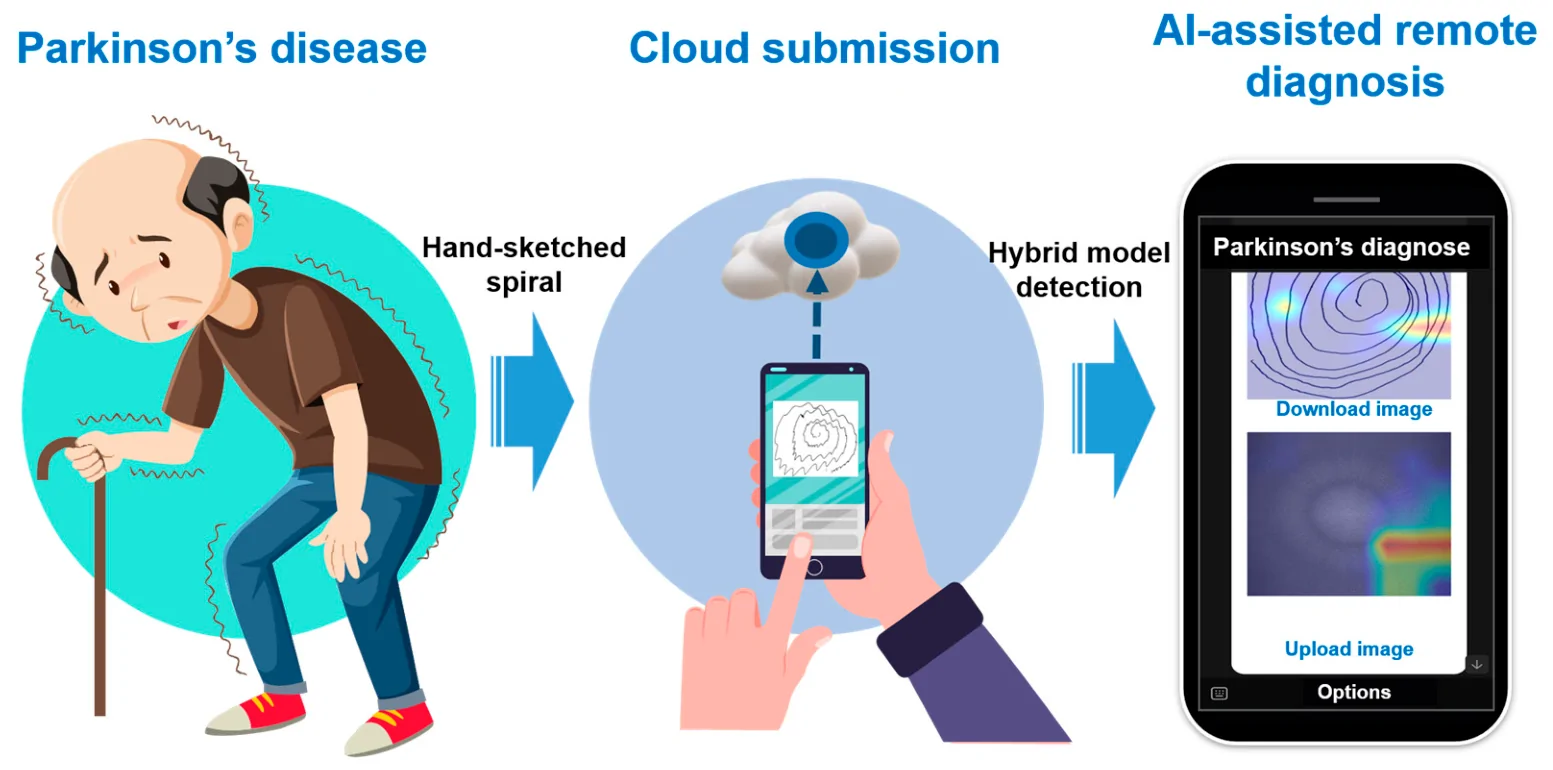
Schematic representation of the LINE-based proof-of-concept workflow for remote handwriting image submission and automated model inference.

**Figure 5:**
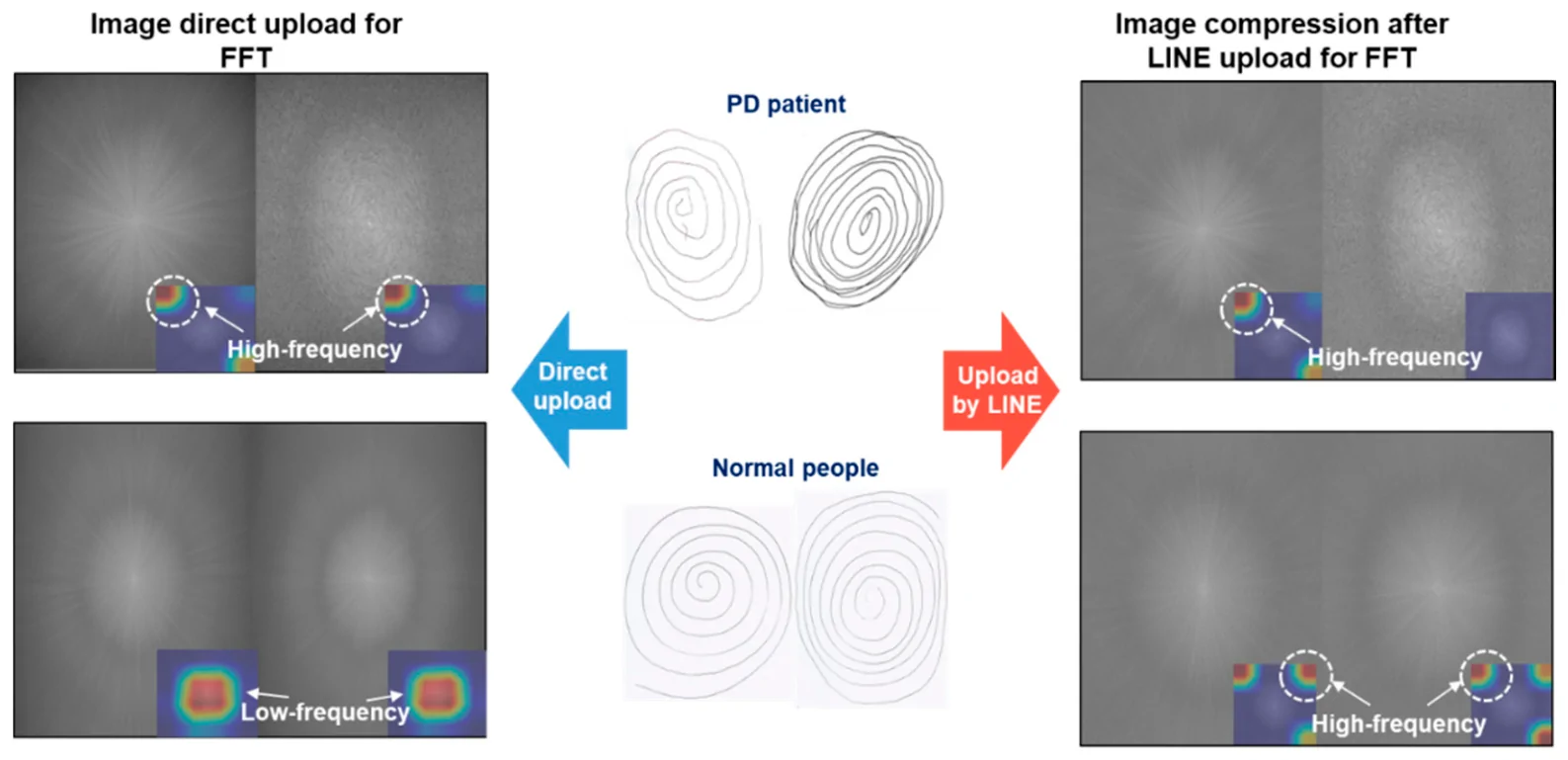
Effect of LINE transmission on spiral images drawn on A4 paper. Compression during the LINE transmission workflow altered image texture and frequency-domain components and was associated with reduced specificity for identifying non-PD individuals.

**Figure 6:**
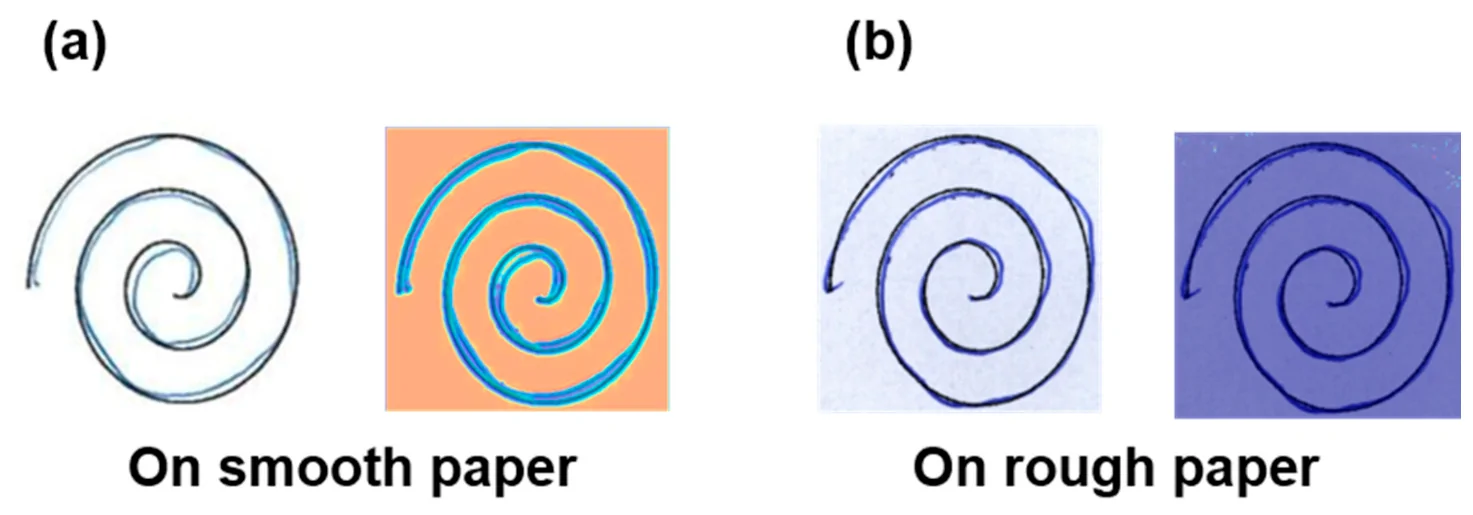
Comparison of background paper texture of (**a**) on smooth paper and (**b**) on rough paper.

**Table 1:** Performance comparison of the proposed dual-branch network and both single-branch networks.

Task Type	Applied Model	Accuracy (%)	F1-Score (%)	Precision (%)	Sensitivity (%)	Specificity (%)
Circle task	FreqNet only	81.98 ± 9.69	81.43 ± 10.37	84.64 ± 8.12	82.14 ± 9.56	74.29 ± 21.19
	SpatialNet only	84.95 ± 9.11	84.59 ± 9.42	88.67 ± 6.58	85.71 ± 8.75	71.43 ± 17.50
	FusionNet	86.48 ± 12.36	85.86 ± 13.08	88.67 ± 11.31	85.95 ± 12.69	88.57 ± 18.63
Meander task	FreqNet only	88.64 ± 1.84	88.61 ± 1.84	88.75 ± 1.81	88.77 ± 1.91	86.39 ± 4.11
	SpatialNet only	78.79 ± 3.09	78.16 ± 3.69	84.49 ± 1.32	79.93 ± 3.48	59.86 ± 6.96
	FusionNet	91.68 ± 1.65	91.66 ± 1.65	91.68 ± 1.66	91.85 ± 1.89	90.02 ± 1.48
Spiral task	FreqNet only	86.36 ± 4.88	86.35 ± 4.87	86.46 ± 4.91	86.55 ± 4.80	85.03 ± 6.72
	SpatialNet only	78.79 ± 3.09	78.16 ± 3.69	84.49 ± 1.32	79.93 ± 3.48	59.86 ± 6.96
	FusionNet	90.91 ± 2.81	90.87 ± 2.84	91.14 ± 2.67	91.07 ± 2.97	89.3 ± 3.54

**Table 2:** Difference between the SVM and FusionNet models with respect to classification performance.

Task Type	Applied Model	Accuracy (%)	F1-Score (%)	Precision (%)	Sensitivity (%)	Specificity (%)
Circle task	FreqNet (SVM)	75.93 ± 9.11	78.69 ± 6.70	68.45 ± 7.62	93.33 ± 9.13	60.00 ± 18.63
	SpatialNet (SVM)	86.26 ± 12.71	86.46 ± 13.21	81.31 ± 14.46	93.33 ± 14.91	80.00 ± 16.29
	FusionNet	86.48 ± 12.36	85.86 ± 13.08	88.67 ± 11.31	85.95 ± 12.69	88.57 ± 18.63
Meander task	FreqNet (SVM)	88.66 ± 4.78	88.59 ± 3.97	85.77 ± 6.74	92.02 ± 4.80	85.54 ± 9.24
	SpatialNet (SVM)	88.26 ± 2.79	88.13 ± 2.74	84.2 ± 5.74	92.76 ± 3.31	84.35 ± 6.39
	FusionNet	91.68 ± 1.65	91.66 ± 1.65	91.68 ± 1.66	91.85 ± 1.89	90.02 ± 1.48
Spiral task	FreqNet (SVM)	85.23 ± 3.39	84.98 ± 3.56	81.34 ± 5.07	89.39 ± 6.41	81.33 ± 7.27
	SpatialNet (SVM)	90.90 ± 2.51	90.71 ± 2.74	86.77 ± 3.24	95.09 ± 3.45	87.13 ± 3.24
	FusionNet	90.91 ± 2.81	90.87 ± 2.84	91.14 ± 2.67	91.07 ± 2.97	89.3 ± 3.54

**Table 3:** Representative handwriting-based PD detection approaches.

Study	Dataset/Participants	Drawing Task	Proposed Model	Key Features	Performance
Alissa et al. (2022) [16]	87 participants: 58 PD, 29 health control (HC)	Wire cube, spiral pentagon	A compact CNN architecture	Detecting deviations from patients’ drawing movements	Best CNN: accuracy 93.53% in cross-validation; test accuracy 95.02%
Li et al. (2022) [12]	86 participants: 43 PD, 43 HC	Template-free Archimedean spiral	A Continuous Convolution Network (CC-Net)	Reduced pooling to retain detailed visual information	Accuracy ~89.3%; MCC ~0.733; mean AUC ~0.934
Ali et al. (2025) [13]	NewHandPD, 66 participants: 31 PD, 35 HC; External test: HandPD, 92 participants: 74 PD, 18 HC	Spiral and meander	χ^2^-refined deep features with a genetically optimized neural network (ChiGa-Net)	Statistical feature refinement with neural networks optimized using a genetic algorithm	External spiral: balanced accuracy 92.56%, sensitivity 85.13%, specificity 100%; external meander: 83.48%, 89.18%, 77.77%, respectively
FusionNet (proposed)	NewHandPD, 66 participants: 31 PD, 35 HC	Circle, meander, spiral	A spatial and frequency dual-branch transformer architecture	Reciprocal fusion of spatial and log-magnitude FFT features	Best observed (Meander): accuracy 91.68%, F1-score 91.66%, sensitivity 91.85%, specificity 90.02%

## Data Availability

The source code and implementation are publicly available in the official GitHub repository: https://github.com/217wei/FusionNet-PD-Handwriting (accessed on 11 August 2026).

## Supplementary Materials

The following supporting information can be downloaded at: https://www.mdpi.com/article/10.3390/bioengineering13101101/s1, Video S1: operational video; Video S2: LINE-based interface to hospital registration system.

## Abbreviations

The following abbreviations are used in this manuscript:

AD	Alzheimer’s Disease
AI	Artificial Intelligence
BCA	Bidirectional Cross-Attention
CNN	Convolutional Neural Network
DConv	Depth-wise Convolution
FFT	Fast Fourier Transform
FN	False Negative
FP	False Positive
GAP	Global Average Pooling
GDFN	Gated Depth-wise Convolutional Feed-forward Network
GFLOP	Giga Floating-point Operation
GELU	Gaussian Error Linear Unit
GMACs	Giga multiply-accumulate operations per second
Grad-CAM	Gradient-weighted Class Activation Mapping
HC	Healthy Control
MDTA	Multi-DConv Head Transposed Self-Attention
MRI	Magnetic Resonance Imaging
MSA	Multiple System Atrophy
PD	Parkinson’s Disease
PET	Positron Emission Tomography
RBF	Radial Basis Function
SPECT	Single-Photon Emission Computed Tomography
SQL	Structured Query Language
SVM	Support Vector Machine
TN	True Negative
TP	True Positive

## References

[1] Zhang X., Baht G.S., Huang R., Chen Y.H., Molitoris K.H., Miller S.E., Kraus V.B. (2022). Rejuvenation of neutrophils and their extracellular vesicles is associated with enhanced aged fracture healing. Aging Cell.

[2] Chen Y.H., Byers Kraus V., Chang Y.S., Lin T.M., Lee H.Y., Chang C.K., Kuo T.T., Chen J.H., Chang C.C. (2026). Dipeptidyl peptidase-4 inhibitors are associated with a lower risk of osteoarthritis in patients with type 2 diabetes. Ther. Adv. Musculoskelet. Dis..

[3] Hsu L.J., Chang Y., Liao C.J., Wang C.Y., Gupta S., Lee C.Y., Lai Y.T., Tai N.H. (2026). Integration of hollow nanodices Ni(OH)_2_ and carbon nanotubes for exceptional noninvasive salivary glucose biosensor. J. Alloys Compd..

[4] Chen Y.H., Chung C.H., Liu F.C., Chien W.C. (2026). Increasing Cancer Risk in Patients with Nephrotic Syndrome: A Nationwide Population-based Study in Taiwan. J. Med. Sci..

[5] Dorsey E.R., Sherer T., Okun M.S., Bloem B.R. (2018). The Emerging Evidence of the Parkinson Pandemic. J. Park. Dis..

[6] Church F.C., Rau J.C., Brotini S. (2026). Parkinson’s Disease: Pathophysiology, Treatment Strategies, Wellness Approaches, and Obstacles/Paths Forward. NeuroSci.

[7] Räty V., Kuusimäki T., Majuri J., Vahlberg T., Gardberg M., Noponen T., Seppänen M., Tolppanen A.M., Kaasinen V. (2025). Stability and Accuracy of a Diagnosis of Parkinson Disease Over 10 Years. Neurology.

[8] Zampogna A., Suppa A., Patera M., Cavallieri F., Bove F., Fraix V., Castrioto A., Schmitt E., Pelissier P., Chabardes S. (2025). Resting and action tremor in Parkinson’s disease: Pathophysiological insights from long-term STN-DBS. npj Park. Dis..

[9] Shevelkova V., Mattocks C., Lafortune L. (2023). Efforts to address the Sustainable Development Goals in older populations: A scoping review. BMC Public Health.

[10] Calabresi P., Mechelli A., Natale G., Volpicelli-Daley L., Di Lazzaro G., Ghiglieri V. (2023). Alpha-synuclein in Parkinson’s disease and other synucleinopathies: From overt neurodegeneration back to early synaptic dysfunction. Cell Death Dis..

[11] Bidesi N.S.R., Vang Andersen I., Windhorst A.D., Shalgunov V., Herth M.M. (2021). The role of neuroimaging in Parkinson’s disease. J. Neurochem..

[12] Li Z., Yang J., Wang Y., Cai M., Liu X., Lu K. (2022). Early diagnosis of Parkinson’s disease using Continuous Convolution Network: Handwriting recognition based on off-line hand drawing without template. J. Biomed. Inform..

[13] Ali L., Leung M.-F., Khan M.A., Nour R., Imrana Y., Vasilakos A.V. (2025). ChiGa-Net: A genetically optimized neural network with refined deeply extracted features using χ2 statistical score for trustworthy Parkinson’s disease detection. Neurocomputing.

[14] Hope T.M.H., Bowman H., Leff A.P., Price C.J. (2025). Deep convolutional neural networks outperform vanilla machine learning when predicting language outcomes after stroke. NeuroImage Clin..

[15] Turyamuhaki N., Sameena A., Ahishakiye E. (2026). Deep ensemble learning with convolutional and transformer models for brain stroke classification. Discov. Artif. Intell..

[16] Alissa M., Lones M.A., Cosgrove J., Alty J.E., Jamieson S., Smith S.L., Vallejo M. (2022). Parkinson’s disease diagnosis using convolutional neural networks and figure-copying tasks. Neural Comput. Appl..

[17] Pereira C.R., Weber S.A.T., Hook C., Rosa G.H., Papa J.P. Deep Learning-Aided Parkinson’s Disease Diagnosis from Handwritten Dynamics. Proceedings of the 2016 29th SIBGRAPI Conference on Graphics, Patterns and Images (SIBGRAPI).

[18] Mai J., Gao C., Bao J. (2025). Domain Generalization Through Data Augmentation: A Survey of Methods, Applications, and Challenges. Mathematics.

[19] Rao Y., Zhao W., Zhu Z., Lu J., Zhou J. (2021). Global Filter Networks for Image Classification. arXiv.

[20] Hao P.-Y., Chiang J.-H., Chen Y.-D. (2022). Possibilistic classification by support vector networks. Neural Netw..

[21] Zamir S.W., Arora A., Khan S., Hayat M., Khan F.S., Yang M. Restormer: Efficient Transformer for High-Resolution Image Restoration. Proceedings of the 2022 IEEE/CVF Conference on Computer Vision and Pattern Recognition (CVPR).

[22] Vasconcelos J.C.S., Cordeiro G.M. (2026). An Error Function-Based Regression Model for Depressogenic Reasoning Data Analysis. Stats.

[23] Loshchilov I., Hutter F. (2017). Decoupled weight decay regularization. arXiv.

[24] Loshchilov I., Hutter F. (2016). Sgdr: Stochastic gradient descent with warm restarts. arXiv.

[25] 2022 AIGO Competition.

[26] Geirhos R., Jacobsen J.-H., Michaelis C., Zemel R., Brendel W., Bethge M., Wichmann F.A. (2020). Shortcut learning in deep neural networks. Nat. Mach. Intell..

[27] Hill B.G., Koback F.L., Schilling P.L. (2024). The risk of shortcutting in deep learning algorithms for medical imaging research. Sci. Rep..

[28] Ong Ly C., Unnikrishnan B., Tadic T., Patel T., Duhamel J., Kandel S., Moayedi Y., Brudno M., Hope A., Ross H. (2024). Shortcut learning in medical AI hinders generalization: Method for estimating AI model generalization without external data. npj Digit. Med..

